# Neonatal Handling Positively Modulates Anxiety, Sensorimotor Gating, Working Memory, and Cortico-Hippocampal Neuroplastic Adaptations in Two Genetically Selected Rat Strains Differing in Emotional and Cognitive Traits

**DOI:** 10.3390/brainsci15080776

**Published:** 2025-07-22

**Authors:** Cristóbal Río-Álamos, Maria P. Serra, Francesco Sanna, Maria A. Piludu, Marianna Boi, Toni Cañete, Daniel Sampedro-Viana, Ignasi Oliveras, Adolf Tobeña, Maria G. Corda, Osvaldo Giorgi, Alberto Fernández-Teruel, Marina Quartu

**Affiliations:** 1 Department of Psychology, School of Medicine, Austral University of Chile, Valdivia 5091000, Chile; cristobal.delrio@uach.cl; 2 Department of Psychiatry & Forensic Medicine, Medical Psychology Unit, School of Medicine & Institute of Neurosciences, Autonomous University of Barcelona, 08193 Barcelona, Spain; antoni.canete@uab.cat (T.C.); daniel.sampedro@uab.cat (D.S.-V.); ignasioliverasp@gmail.com (I.O.); adolf.tobena@uab.cat (A.T.); 3 Department of Biomedical Sciences, Section of Cytomorphology, University of Cagliari, Cittadella Universitaria di Monserrato, 09042 Monserrato, CA, Italy; mpserra@unica.it (M.P.S.); marianna.boi@unica.it (M.B.); 4 Department of Life and Environmental Sciences, Section of Pharmaceutical, Pharmacological and Nutraceutical Sciences, University of Cagliari, 09042 Monserrato, CA, Italymariagiuseppacorda53@gmail.com (M.G.C.); giorgi@unica.it (O.G.); 5 Department of Medicine, Faculty of Medicine and Health Sciences, International University of Catalunya, 08195 Barcelona, Spain

**Keywords:** anxiety, stress, prepulse inhibition, spatial working memory, Roman high- and low-avoidance rats, neonatal handling, BDNF, trkB, PSA-NCAM, prefrontal cortex, hippocampus

## Abstract

**Background/Objectives**: The bidirectional selection of the Roman low- (RLA) and Roman high-avoidance (RHA) rat strains for extremely slow vs. very rapid acquisition of the two-way (shuttle-box) avoidance response has generated two divergent phenotypic profiles: RHA rats exhibit a behavioural pattern and gene expression profile in the frontal cortex and hippocampus (HPC) that are relevant to social and attentional/cognitive schizophrenia-linked symptoms; on the other hand, RLA rats display phenotypic traits linked to increased anxiety and sensitivity to stress-induced depression-like behaviours. The present studies aimed to evaluate the enduring and potentially positive effects of neonatal handling-stimulation (NH) on the traits differentiating these two strains of rats. **Methods**: We evaluated the effects of NH on anxious behaviour, prepulse inhibition of startle (PPI), spatial working memory, and hormone responses to stress in adult rats of both strains. Furthermore, given the proposed involvement of neuronal/synaptic plasticity and neurotrophic factors in the development of anxiety, stress, depression, and schizophrenia-related symptoms, using Western blot (WB) we assessed the effects of NH on the content of brain-derived neurotrophic factor (BDNF), its trkB receptor and Polysialilated-Neural Cell Adhesion Molecule (PSA-NCAM), in the prefrontal cortex (PFC), anterior cingulate cortex (ACg), ventral (vHPC), and dorsal (dHPC) hippocampus of adult rats from both strains. **Results**: NH increased novelty-induced exploration and reduced anxiety, particularly in RLA rats, attenuated the stress-induced increment in corticosterone and prolactin plasma levels, and improved PPI and spatial working memory in RHA rats. These effects correlated to long-lasting increases of BDNF and PSA-NCAM content in PFC, ACg, and vHPC. **Conclusions**: Collectively, these findings show enduring and distinct NH effects on neuroendocrine and behavioural and cognitive processes in both rat strains, which may be linked to neuroplastic and synaptic changes in the frontal cortex and/or hippocampus.

## 1. Introduction

The Roman low-avoidance (RLA) and high-avoidance (RHA) rat strains are psychogenetically selected for, respectively, extremely slow versus very rapid acquisition of two-way active (shuttle-box) avoidance learning [1,2,3,4]. This genetic selection has led to marked and stable divergences in various neurobehavioural phenotypes/traits between these two rat strains. When exposed to anxiogenic or stressful situations, RLA rats display a passive and reactive coping style, and are more anxious, fearful, sensitive to frustration, sensitive to stress, and prone to depression compared to RHA rats (e.g., [2,3,4,5,6,7,8,9]). In contrast, relative to RLA rats and other unselected rat strains, RHA rats present a proactive coping style, resistance to stress and to depression-like symptoms, increased impulsivity and novelty-seeking behaviour, decreased social behaviour and impaired attention, sensorimotor gating and cognitive deficits, as well as enhanced mesolimbic dopamine-mediated behavioural sensitization following chronic administration of morphine or psychostimulants such as amphetamine and cocaine, and vulnerability to drug addiction (e.g., [2,3,4,10,11,12,13]).

Since the establishment of the first colony in 1965 [1], a large body of evidence has accumulated highlighting neurochemical, molecular, pharmacological, and gene-expression differences between RHA and RLA rats. In summary, compared to RLAs, the RHA rats present alterations of pre- and postsynaptic markers as well as trophic factors in the PFC and/or hippocampus (HPC) (e.g., neuregulin1, homer1, synaptophysin, brain-derived neurotrophic factor-BDNF), which are associated with dysfunctional glutamatergic and dopaminergic neural systems, altered PFC maturation, and psychiatric disorders (e.g., [2,3,4,14,15,16], and references therein). In addition, RHA rats exhibit increased density of D1 subtype dopamine (DA) receptors and NMDA2B receptors, along with enhanced 5-HT2A receptor density, in the PFC. In contrast, they show a dramatic deficit of mGlu2 receptors in PFC, HPC, and striatum (reviewed by [2]). These findings are consistent with experimental evidence indicating a reduced function and volume of the PFC and HPC in RHA rats (e.g., [2,17,18]), and an enhanced density of immature dendritic spines in the PFC of RHA rats [2]. These molecular/synaptic marker profiles, along with the reported changes in the mesolimbic and mesocortical DA systems [2,4], strongly suggest that RHA rats have an immature cortical structure/function, involving the PFC and, possibly, the HPC. This assumption is consistent with their behavioural and cognitive profile and with features relevant to schizophrenia and drug addiction [2,3,4,16,17,18].

RLA rats show alterations in central serotonin and GABA-A/benzodiazepine neurotransmission (reviewed by [2,19]). They also exhibit increased function in the amygdala [2,3,17,18,20] and enhanced activity in the (amygdala–)hypothalamus–pituitary–adrenal axis (e.g., [2,3,4,5,6,8,9,19,21], and references therein). Along with the effects of GABA-A/benzodiazepine and serotoninergic drugs on their anxiety- and depression-related profiles (e.g., [3,4]; see review in [2] and Supplementary Tables S1 and S2 therein), the above-mentioned factors may be linked to the neurobiological mechanisms underlying the anxious, behaviourally inhibited, and stress- and depression-sensitive traits observed in RLA rats.

To summarize, the neurobehavioral evidence presented supports the validity of RLA rats as a model for studying anxiety, fearfulness, sensitivity to stress, and stress-induced depression-like symptoms. On the other hand, RHA rats may be a translational model for exploring developmental, neural, behavioural, and cognitive features relevant to schizophrenia and concomitant vulnerability to drug-addiction [2,3,4,9,21].

Neurotrophins, particularly BDNF, are considered to play a key role in the pathophysiology of depression, stress, and schizophrenia. Inadequate neurotrophic support in early developmental stages can lead to several consequences related to disorganization of the brain structure. Specifically, it is believed that a decreased BDNF/trkB-mediated cell support may result in reduced neurogenesis, neuronal atrophy and glial cell loss in critical brain areas [22,23,24,25], and these abnormal processes have been related to increased vulnerability to depression and schizophrenia symptoms. Regarding depression, the chronic treatment with antidepressants has been reported to increase BDNF expression in the rat HPC [26,27], while BDNF showed anti-depressant actions in learned helplessness-related paradigms, such as the forced swimming test [28]. Decreased central BDNF content has been observed in rodent models of stress-induced depressive symptoms [27,29,30], indicating a downregulation of the trkB signalling pathway [31]. Interestingly, the above evidence is in line with findings from studies comparing RLA with RHA rats. Indeed, the stress-sensitive and depression-prone RLA rats show lower BDNF and trkB protein content in the hippocampus compared to RHA rats, consistent with the greater susceptibility of RLA rats to stress-related depression-like symptoms ([4,14] and references therein). Moreover, acute swimming stress was found to decrease BDNF levels in the vHPC and increase them in the dHPC of RLA rats whereas it was devoid of effect on BDNF content in RHAs [14]. More recent studies have shown that the direction and/or magnitude of these effects may vary depending on the intensity of the stressor [15].

The neurotrophic hypothesis of schizophrenia posits that the alterations observed in the brains of individuals with schizophrenia stem from disruptions in developmental processes involving BDNF. Numerous studies in humans and animal models have investigated the role of BDNF and its receptor trkB in this disorder [22,32]. Thus, it has been shown that patients with schizophrenia have decreased concentrations of BDNF in HPC and PFC [33,34]. Variations in BDNF may lead to changes in the brains of patients with schizophrenia, including a reduction in the volume of frontal grey matter and an increase in the volume of lateral ventricles and sulcal cerebrospinal fluid (CSF) [35]. Additionally, BDNF has been explored as a potential biomarker for diagnosing and evaluating cognitive aspects of schizophrenia [25,36,37,38], and the val(66)Met polymorphism of the BDNF gene may be associated with risk of schizophrenia symptoms [39,40]. Notably, reduced BDNF mRNA levels have been reported in rats with neonatally induced lesions of the vHPC (a neurodevelopmental model that mimics features associated with schizophrenia) [41,42]. Accordingly, the administration of MK801 (dizocilpine), a glutamatergic NMDA receptor antagonist that produces positive, negative, and cognitive schizophrenia-like symptoms, reduced the expression of BDNF in the HPC [43]. In this context, differences in fronto-cortical and hippocampal *Bdnf* expression and BDNF protein levels (and other pre-/postsynaptic markers; [2,16]), as well as in the behavioural effects of MK801, have been reported in RHA vs. RLA rats (see review by [2]).

The neonatal handling (NH) intervention (commonly administered to rats or mice during the first 2–3 postnatal weeks), induces lifelong improvements in emotional behaviour and coping with stress, and enhances attentional and cognitive efficiency (e.g., [44,45,46]). Moreover, the NH treatment enduringly reduces hormone responses to stress, promotes neuroplasticity and neurogenesis, improves HPC function, prevents neurodegeneration in the HPC, and affects various neurotransmitter systems (e.g., [44,47,48,49,50,51,52,53,54,55]). Importantly, most of these NH effects have similarly been reported in Roman rats (e.g., [12,18,44]).

This work was therefore undertaken to investigate the long-lasting impact of NH on anxiety-/depression- and schizophrenia-related behaviours/responses in RHA and RLA rats. To this purpose, we assessed (1) novelty-related behaviours, (2) stress-induced corticosterone and prolactin responses, (3) baseline startle response, (4) prepulse inhibition of the acoustic startle response (PPI), and (5) spatial working memory. Additionally, we performed Western blot assays in samples from the PFC, ACg, vHPC, and dHPC of both Roman strains to determine whether those effects were associated with changes in the protein levels of BDNF, trkB, and PSA-NCAM.

## 2. Material and Methods

### 2.1. Animals and Neonatal Handling (NH) Treatment

**Figure 1:**
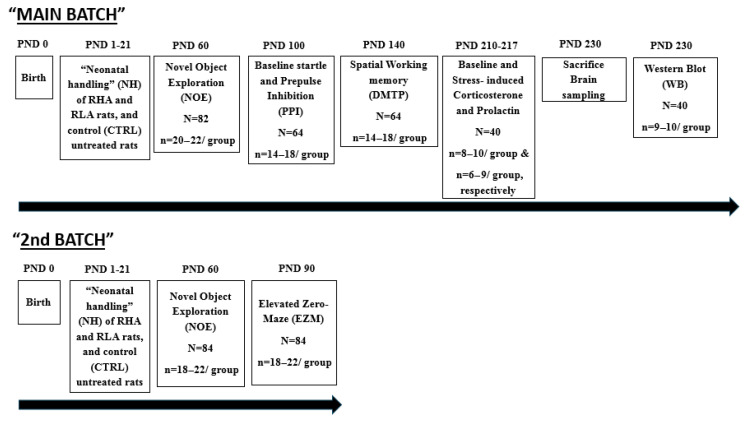
Overview of the experimental design and timeline (see Section 2). The study was carried out with male rats of the RHA and RLA strains, receiving neonatal handling (NH) or no treatment (controls, CTRL). “N”, means the total number of rats used in a given experiment or test/measure; “n”, means the number of rats (or range) per experimental group for a particular experiment/test. In some cases, such as the stress-induced hormonal (corticosterone, prolactin) responses, or in WB assays, some rats were excluded from the final analyses because they were outliers (i.e., they had values < or > 2 SD from the respective group mean).

The studies were carried out with 166 male RHA and RLA rats (approximately half/strain). They were experimentally naïve and came from our own colony (established in 1996) of inbred Roman rats. The neonatal handling (NH) intervention started the day after birth (postnatal day 1, PND1), and was administered every day until PND21 (the weaning day; see Figure 1). Every day (from PND1 to PND21) in the morning (9:30 h) and evening (17:00 h) the mother was separated from the pups and placed in a different cage, and then each pup was individually placed in a small plastic cage (25 × 25 × 15 cm), with some paper towel in its bottom, in a different room (temperature 22–24 °C), where it remained for 8 min (see [12,18]). The experimenter handled/stroked very gently (without gloves) each individual pup for 3–4 s at the beginning, at 4 min, and at the end of the 8-min separation period. After this, each pup was returned to its home cage with the rest of the litter and the dam. During this three-week period, non-handled (control; CTRL) rats received no manipulation, except for weekly cage cleaning. In all the following studies and tests/tasks, control (unhandled) and NH-treated groups had rats from 6–10 different litters. On the weaning day (PND21), all rats were housed in pairs of the same experimental group in standard cages, with free access to water and food, and a controlled 12 h light–dark cycle (light on at 08:00 h), temperature (22 + 2 °C), and humidity (50–70%). Two separate cohorts of rats, the “MAIN BATCH” and the “2nd BATCH”, each one going through separate experimental procedures, were used. Figure 1 represents an overview of the timeline of the experimental procedures.

The animal study protocol conformed with the institutional guidelines of the Committee for Animal Experimentation of the Universidad Autónoma de Barcelona (protocol reference number 875-CEEAH, approved on 27 July 2017)  and was authorized by the spanish Ministerio de Ciencia e Innovación (reference No. PID2020-114697GB-I00, 1 January 2021)

### 2.2. Behavioural and Hormonal Measures

#### 2.2.1. Novel Object Exploration (NOE) Test of Anxiety-Related Behaviour

The NOE test was conducted when the rats were 60 days old to assess their response to novelty-induced behavioural inhibition/disinhibition (Figure 1). This test involved evaluating the rats’ exploratory behaviour when a novel object was introduced in their home cage. We have shown that this test serves as an effective index of the anxiolytic effects of NH intervention. Specifically, rats treated with NH show enhanced exploration of the novel object, and this is associated with the NH-anti-anxiety effects in other validated models of anxiety (e.g., [12,18]).

One hour before starting the testing procedure, the food was removed from the home cage, and only four pellets were left. Then, the home cage was pulled from the rack about 20 cm, to facilitate direct observation. The novel object, a graphite pencil, was then introduced vertically through the cage grid cover until contacting the cage bedding, and the two rats within each cage were simultaneously observed and scored for 3 min by a blinded trained observer. The measures taken during the 3 min test were NOE-L, latency to explore, i.e., the time taken until the rat approaches (to < 1 cm) and explores the novel object for the first time; NOE-T, the total duration of pencil exploration (NOE-T). The experimenter/observer stood 50 cm from the front of the cage to ensure accurate observation.

#### 2.2.2. Elevated Zero-Maze (EZM) Test of Anxiety

An independent batch of rats (“2nd BATCH”, indicated with “(#)” in the Section 3 and Table 1), that were also tested in the NOE when 60 days old, were evaluated for anxiety in the elevated circular (zero-)maze (EZM) at 90 days of age (Figure 1). The maze was built of black plywood, was elevated 65 cm above the floor, having the shape of a round corridor (10 cm width; 105 cm diameter), and having two open (opposite) and two enclosed (opposite) sections. The enclosed sections were surrounded/protected by 40 cm height walls, and the maze was placed in a testing room with the floor, walls, and roof painted black. The observation room was dimly illuminated with red fluorescent light. Each 5 min testing trial started by placing the rat in an enclosed section of the EZM, and behaviour was videotaped and scored outside the room watching a TV monitor. A well-trained experimenter, who was blinded to the rat’s experimental group, scored the “time spent into open sections” (EZM-T) and the “number of head-dips” (EZM-HD) a rat did through the borders of the maze open sections (these are the two most sensitive measures according to previous studies; see [18]).

#### 2.2.3. Baseline Startle and Prepulse Inhibition of the Acoustic Startle Response (Sensorimotor Gating, PPI)

This task was administered when rats were 100 days old (Figure 1), according to our previously published method [11]. Four sound attenuated chambers (SR-Lab Startle Response System, San Diego Instruments, San Diego, CA, USA) were used. Each box had a Plexiglas cylinder placed onto a platform. Each rat is enclosed in the cylinder for a testing session. The platform detects the strength of each startle response made by the rat. An accelerometer transduces the strength of the rat’s startle into a voltage, which is amplified. The information is digitized and is saved into a computer in numeric files than can be analysed. Two speakers, placed 15 cm away from each side of the cylinder where the rat is enclosed, provide the acoustic stimuli. Background noise is constantly provided by a white noise generator, and a constant lit from a 10 W lamp provides internal illumination to each startle box.

Each test session consisted of

1. Five-minute habituation to the startle chamber (only background noise, 55 dB).
2. Ten “pulse-alone” trials (105 dB, 40 ms), to allow some habituation of startle and more stable “baseline startle” responses.
3. Then, six different trial types were administered 10 times in a random order (for a total of 60 trials). These six trial types were as follows: **(i)** Pulse-alone (105 dB, 40 ms), “baseline startle” trials, from which the percentage prepulse inhibition (%PPI) is calculated. **(ii–v)** Prepulse stimuli (20 ms) of four different intensities (65, 70, 75, 80 dB), plus a 100 ms interval followed by the acoustic startle stimulus of 105 dB (40 ms). **(vi)** Background noise (55 dB), i.e., no stimulus trials.

The session ended with five pulse-alone trials (105 dB, 40 ms). Throughout the entire session, the inter-trial interval was 10–20 s (mean = 15 s). The magnitude of each startle response was recorded as the average startle values during 200 ms after the onset of the 105 dB pulse (see [20]).

At each prepulse intensity, the %PPI was calculated by the formula [11]

%PPI = 100 − (startle amplitude on prepulse trial/startle amplitude on pulse trial) × 100

The 14–18 rats/group for the PPI and the following (DMTP) task were randomly selected from the rats that underwent the NOE test.

#### 2.2.4. Morris Water Maze: Delayed Matching-to-Place Task (DMTP) of Spatial Working Memory

Rats were 140 days old when the DMTP task started (Figure 1).

**Figure 2:**
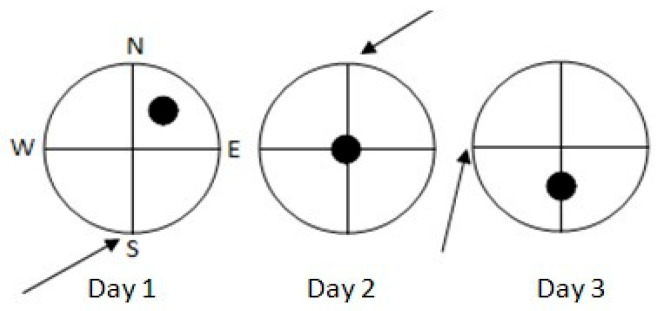
Starting positions (arrows) for both trials of each of the three training days on the DMTP (delayed-matching-to place; spatial working memory) task. Black circles indicate the position of the submerged platform across the three days of training.

We used a circular pool of 150 cm diameter (60 cm height), filled with water (22–24 °C) to a depth of 30 cm. There were no local cues within the pool nor in its walls. Four equally spaced points (N, S, E, W) around the pool wall served as starting positions (Figure 2).

For each swimming navigation trial, each individual rat had to swim for a maximum of 90 s or until it located (and jumped onto) a circular platform (15 cm diameter, 28 cm height) that was submerged 2 cm below the water surface and was not visible for the animals. The rat was gently guided to the platform by the experimenter if it did not find the hidden platform within 90 s. Then, the rat remained on the platform for 15 s and was placed in an individual cage for another 15 s, after which the second trial started from the same starting position. Each rat was administered two trials/day (intertial interval 30 s) for three consecutive days. Trial 1 (T1) was the “sample” trial and Trial 2 (T2) was the retention trial. Within each training day, the platform and starting positions were constant, but changed from day to day (see Figure 2).

Across the three training days, various constant distal cues were present in the room walls and benches, which were visible from the pool. The main parameter was the distance each rat travelled to reach the platform in T1 minus the distance travelled in T2 averaged for the three testing days (“T1–T2”). [11].

Each trial was monitored by a video system and tracked by the “Smart v.2.5.14” (PANLAB, Barcelona, Spain) software. [11].

#### 2.2.5. Hormone Measurements

Baseline and post-stress corticosterone and prolactin were measured in rats from the four experimental groups (n = 10/group, randomly selected from the previous task) at 7 months (210 days) of age (Figure 1). Blood samples were obtained between 9:30 and 12:30 h. A tail-nick procedure was used for blood sampling: a 2 mm incision was made at the end of one of the rat’s tail arteries, and 200 microliters of blood were collected in less than 2 min into ice-cold Safe-lock tubes (Eppendorf, Hamburg, Germany). This procedure was first conducted under resting conditions, just taking the rats from their home cage (baseline measurement) and immediately taking the blood sample. Seven days after this baseline measurement, each rat was exposed for 20 min to a novel environment (a 40 × 40 × 40 cm novel cage) in a testing room (illuminated by regular white light), and post-stress blood samples were obtained from the tail (same procedure as above) immediately after the 20 min novelty exposure. Blood samples were centrifuged and the plasma obtained was kept at −80 °C until performing the hormone assays. Enzyme-linked immunosorbent assay (competitive ELISA) determined plasma corticosterone and prolactin levels. EMS Reader MF V.2.9-0 was the reader make used. The reagents used were Corticosterone EIA (Inmunodiagnostic System Ltd., IDS Ltd.; Boldon, UK) and Prolactin Rat ELISA (Demeditec Diagnostics, GmbH; Kiel, Germany). Finally, the results for each hormone were obtained using the Immulite R 1000 (Siemens, Berlin, Germany) apparatus. Rats showing values of >2 SD above or below the group mean (in any of the hormonal measurements) were excluded from analysis.

### 2.3. Brain Sampling and Molecular Measures

When rats were 7.3 months (230 days) old, 11–13 days after the last blood extraction for hormone measurements, they were sedated with inhaled isofluorane 5% and sacrificed (Figure 1). Immediately after sacrifice by guillotine, the brains were rapidly dissected and processed for Western blot (WB). For WB, brains were cooled in dry ice for 15 s, placed in a brain matrix and cut in 2 mm thick coronal slices using the stereotaxic coordinates of the rat brain atlas of Paxinos and Watson [56] as a reference. Bilateral punches (diameter 2.5 mm) of the dorsal hippocampus were taken as described by Palkovits [57]. For each rat, tissue punches from both hemispheres were pooled, rapidly frozen at −80 °C, and homogenized in distilled water containing 2% sodium dodecylsulfate (SDS) (300 μL/100 mg of tissue) and a cocktail of protease inhibitors (cOmplete^™^, Mini Protease Inhibitor Cocktail Tablets, Cat# 11697498001, Roche, Basel, Switzerland).

#### Western Blot (WB) Assay

Total protein concentrations were determined using the Lowry method [58], with bovine serum albumin as a standard. For the analysis, 40 μg of each tissue homogenate (40) was diluted 3:1 in 4× loading buffer (NuPAGE LDS Sample Buffer 4X, Cat# NP0008, Novex by Life Technologies, Carlsbad, CA, USA) and heated to 95 °C for 7 min. Samples were then separated by sodium dodecyl sulphate (SDS)-polyacrilamide gel electrophoresis (SDS-PAGE) using a precast polyacrylamide gradient gel (NuPAGE 4–12% Bis-Tris Gel Midi, Cat# NP0321, Novex by Life Technologies, Carlsbad, CA, USA) in the XCell4 Sure LockTM Midi-Cell chamber (Life Technologies). Molecular weight (mw) standards (Precision Plus Protein Western C Standards, Cat# 161–0376, Bio-Rad, Hercules, CA, USA) and recombinant human BDNF protein (rhBDNF) (Cat# B-257, Alomone Labs, Jerusalem, Israel) were run in parallel. Membranes were blocked in 20 mm Tris base and 137 mm sodium chloride containing 0.1% Tween 20 (TBS-T) and 5% milk powder for 60 min at room temperature. Primary antibodies included rabbit polyclonal antibodies against BDNF [Cat# N-20 sc-546,RRID:AB_63094, Santa Cruz Biotechnology, Heidelberg, Germany] and trkB [Cat# (794) sc-12,RRID:AB_632557, Santa Cruz Biotechnology], both diluted 1:1000, along with a mouse monoclonal antibody against PSA-NCAM (Cat# MAB5324, RRID:AB_95211, Merck Millipore, Darmstadt, Germany) also diluted 1:1000 in TBS containing 5% milk powder and 0.02% sodium azide. Incubations were carried out for two nights at 4 °C. Following TBS/T rinses, membranes were incubated with a peroxidase-conjugated goat anti-rabbit serum (Cat#9169, RRID:AB_258434, Sigma Aldrich, St Louis, MO, USA), diluted 1:10,000 in TBS/T for 60 min, at room temperature. Loading controls were performed using a mouse monoclonal antibody against glyceraldehyde-3-phosphate dehydrogenase (GAPDH) (MAB374, RRID:AB_2107445, EMD Millipore, Darmstadt, Germany), diluted 1:1000, and a peroxidase-conjugated goat anti-mouse serum (AP124P, RRID:AB_90456, Millipore, Darmstadt, Germany), diluted 1:5000. Nonspecific staining was controlled by stripping and incubating the blots with the relevant secondary antiserum. After TBS/T rinse, protein bands were developed using the Western Lightning Plus ECL (Cat# 103001EA, PerkinElmer, Waltham, MA, USA) and visualised by means of ImageQuant LAS-4000 (GE Healthcare, Little Chalfont, UK). The approximate mw and the relative optical density (O.D.) of labelled bands were evaluated by a blinded examiner. The intensity ratio of BDNF-, trkB-, and PSA-NCAM-libelled bands to GAPDH-positive bands was used to compare relative protein expression in the RHA and RLA strains, with the O.D. quantified using Image Studio Lite Software (RRID:SCR_014211, Li-Cor, https://www.licorbio.com/image-studio, accessed on 8 January 2022).

### 2.4. Statistical Analyses

Behavioural measurements and WB data (protein levels) were statistically evaluated using two-factor (2 strain × 2 NH) ANOVA, or Student’s *t*-test for independent samples in some cases in which there were only two experimental groups. When two-way ANOVA revealed statistically significant effects, Duncan’s multiple range test was applied to ascertain differences between groups. This was performed because we had the hypotheses that in both rat strains, the NH intervention would induce reductions in anxiety-related behaviours and stress-hormone responses, improvements of PPI and spatial working memory, and increases in brain protein levels (particularly of BDNF and PSA-NCAM, according to previous literature; e.g., [55,59,60,61]).

To explore associations among behavioural and molecular measures, we conducted Pearson’s correlation coefficients (*r*) and factor analysis techniques (Varimax and Direct Oblimin rotations).

Since factor analysis should include no more than one third of variables in relation to the total number of subjects (N) [62], we established two criteria to reduce the number of variables. First, we performed a varimax-rotated factor analysis (Varimax orthogonal rotation) with all proteins and brain areas, to extract independent (i.e., uncorrelated) factors for these 12 molecular measures. We selected the two protein measures with the highest loadings on each resulting factor for further analysis. These selected proteins were then used in the subsequent factor analysis alongside the chosen behavioural–hormonal variables. Secondly, we identified non-redundant behavioural and stress hormone variables by selecting those measures with the highest theoretical relevance according to previous research and literature. The measures chosen were NOE-T, “Total %PPI” (averaged for the four prepulse intensities), the “T1–T2” spatial working memory index (derived from the DMTP task), and post-stress corticosterone levels, which is a classic measure of stress-induced HPA-axis activation. Using these four behavioural–hormonal variables along with the proteins selected from the varimax-rotated factor analysis, we conducted an exploratory obliquely-rotated (oblimin direct rotation) principal components analysis to examine the associations among protein levels, behaviour/attention/cognition, and post-stress hormone values.

All analyses were carried out using an SPSS statistical package (SPSS Windows, 9.0.1, SPSS Inc.; Chicago, IL, USA), with the significance level set at *p* < 0.05.

## 3. Results

### 3.1. Novel Object Exploration (NOE) and Elevated Zero-Maze (EZM) Tests

In the “MAIN BATCH”, factorial ANOVA on NOE-L and NOE-T results respectively revealed Strain (F(1,78) = 21.2, *p* < 0.001; F(1,78)= 19.7, *p* < 0.001), NH (F(1,78) = 32.3, *p* < 0.001; F(1,78) = 117.3, *p* < 0.001) and “Strain x NH” (F(1,78) = 17.1, *p* < 0.001; F(1,78) = 25.7, *p* < 0.001) effects (Table 1).

In the separate “2nd BATCH” study, the results for both variables were replicated. Thus, the ANOVA results for NOE-L and NOE-T respectively yielded Strain (F(1,80) = 31.5, *p* < 0.001; F(1,80)= 243.3, *p* < 0.001), NH (F(1,80) = 18.4, *p* < 0.001; F(1,80) = 147.8, *p* < 0.001) and the interaction “Strain x NH” (F(1,80) = 17.4, *p* < 0.001; F(1,80) = 55.8, *p* < 0.001) (Table 1).

Additionally, in the same “2nd BATCH” of rats, evaluated in the EZM anxiety test, the factorial ANOVA on EZM-T showed significant Strain (F(1,80) = 8.8, *p* < 0.01) and NH (F(1,80) = 26.3, *p* < 0.001) effects, as well as Strain (F(1,80) = 36.9, *p* < 0.001) and NH (F(1,80) = 6.0, *p* < 0.05) effects on EZM-HD (Table 1).

### 3.2. Baseline Startle Response and Prepulse Inhibition of the Startle Response (PPI)

As for the PPI test, a repeated measures factorial ANOVA (2 strains × 2 treatment × 4 prepulse intensities) performed on the percentage of PPI (%PPI), revealed significant effects of Strain (F(1,60) = 7.1, *p* < 0.01) and interaction “NH x Prepulse intensity” (Sphericity Assumed, F(3,108) = 3.1, *p* = 0.032) (Table 1). In addition, ANOVA showed a “Strain x NH” (F(1,60) = 7.3, *p* < 0.01) effect on “Total %PPI” (Table 1). These results reflect the findings that, relative to RLAs, RHA rats globally exhibited lower %PPI, both across intensities and in “Total %PPI”, and NH increased %PPI in RHA rats (see post hoc Duncan’s multiple range tests in Table 1). There were no ANOVA effects on baseline startle (Table 1), although the expected difference between control (CTRL) rats of both strains was observed (i.e., RLA-CTRL > RHA-CTRL; see Duncan’s test in Table 1).

### 3.3. Delayed Matching-to-Place (DMPT) Task of Spatial Working Memory

ANOVA on the working memory index (“T1–T2”), revealed a significant effect for NH (F(1,60) = 4.4, *p* < 0.05). Post hoc Duncan’s test showed that NH improved the working memory index in RHA rats (Table 1). No other significant ANOVA effects were observed.

In a separate study focusing uniquely on control (CTRL) RLA and RHA rats (indicated with “(**##**)” in Table 1,) the usual difference between the strains, i.e., RLA exhibiting a higher working memory index than their RHA counterpart, was confirmed (see Table 1, and references in its legend).

### 3.4. Baseline and Post-Stress Corticosterone and Prolactin Responses

Two-factor ANOVA did not reveal any significant effects on baseline corticosterone levels. However, it showed significant effects of Strain (F(1,31) = 27.5, *p* < 0.001) and NH (F(1,31) = 5.2, *p* < 0.05) on post-stress corticosterone levels (Table 1). Overall, post-stress corticosterone levels were higher in RLA rats, and NH mainly reduced these levels in RHA rats (see Duncan’s test in Table 1).

Regarding baseline prolactin levels, a significant effect of Strain (F(1,26) = 14.2, *p* < 0.001) was observed, indicating that these levels were higher in RLA rats (Table 1). ANOVA conducted on post-stress prolactin levels revealed significant effects of both Strain (F(1,26) = 52.8, *p* < 0.001) and NH (F(1,26) = 10.7, *p* < 0.01), as post-stress prolactin levels were generally higher in RLA rats, while NH overall reduced these levels, particularly in RLAs (see Duncan’s test in Table 1).

### 3.5. BDNF, trkB, and PSA-NCAM Protein Levels in PFC (Prelimbic/Infralimbic) and ACg of RHA and RLA Rats: Effects of Neonatal Handling

**Figure 3:**
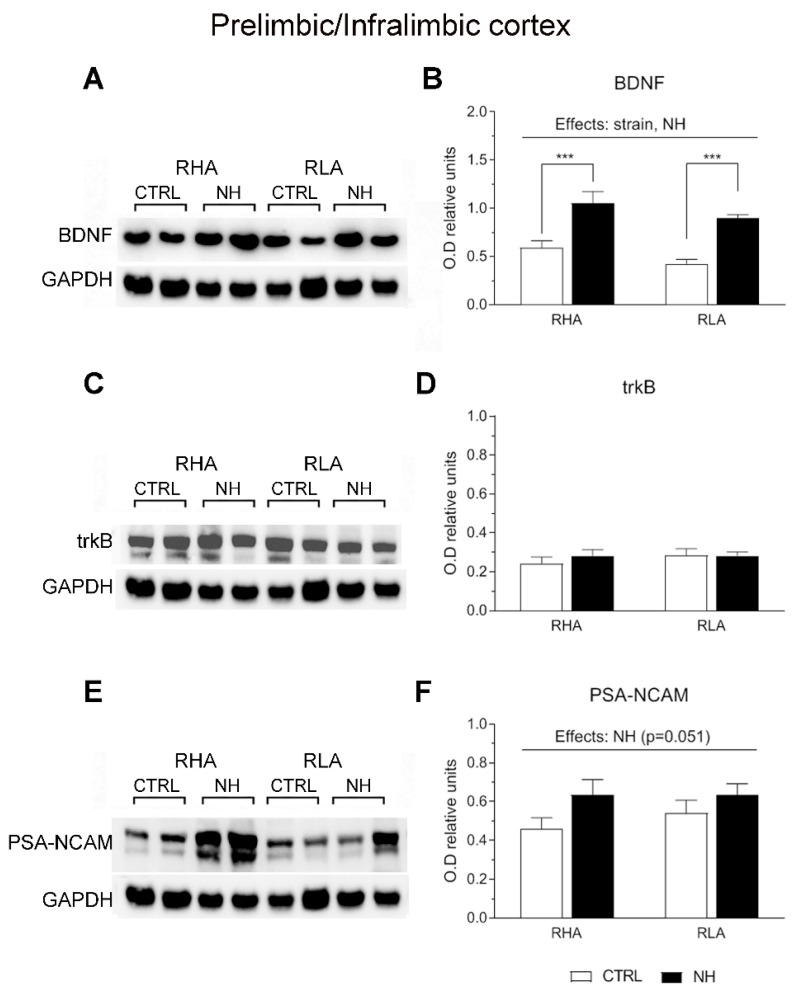
Western blot analysis of the BDNF (**A**,**B**), trkB (**C**,**D**), and the PSA-NCAM (**E**,**F**), in the prefrontal (prelimbic/infralimbic) cortex of the RHA and the RLA rats, either untreated controls (CTRL) or treated with neonatal handling (NH). The values represent the densitometric analysis of the BDNF/GAPDH (**B**), trkB/GAPDH (**D**), and the PSA-NCAM/GAPDH (**F**) band grey optical density (O.D.) ratios. The bars denote the mean ± S.E.M. of 9–10 rats, in each experimental group. ***: *p* < 0.001 (post hoc Duncan’s multiple range test).

**Figure 4:**
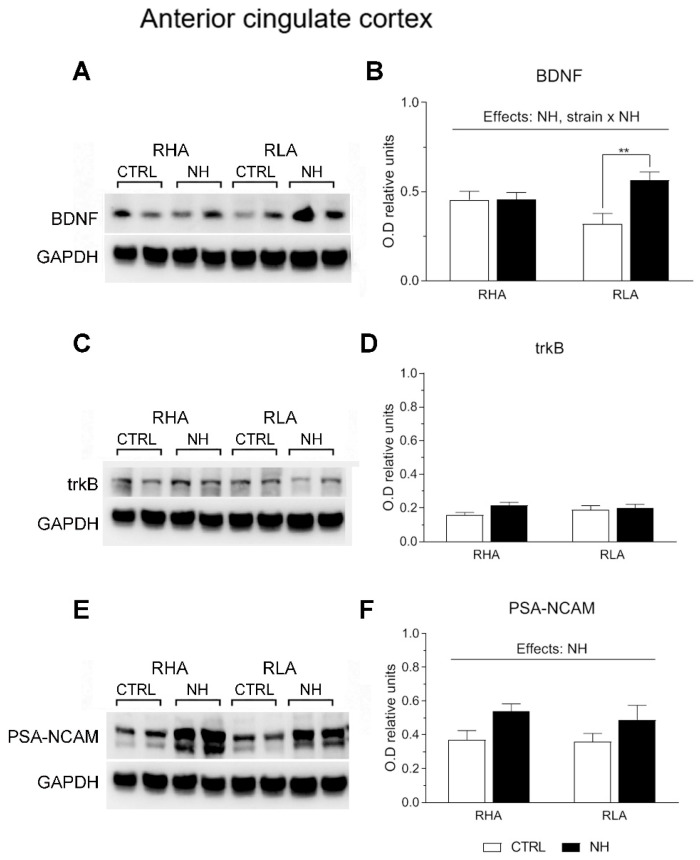
Western blot analysis of the BDNF (**A**,**B**), trkB (**C**,**D**), and the PSA-NCAM (**E**,**F**), in the anterior cingulate cortex of the RHA and the RLA rats, either untreated controls (CTRL) or treated with neonatal handling (NH). The values represent the densitometric analysis of the BDNF/GAPDH (**B**), trkB/GAPDH (**D**), and the PSA-NCAM/GAPDH (**F**) band grey optical density (O.D.) ratios. The bars denote the mean ± S.E.M. of 9–10 rats, in each experimental group. **: *p* < 0.01 (post hoc Duncan’s multiple range test).

Two-factor ANOVA revealed significant effects of Strain on BDNF levels in the PFC (F(1,33) = 4.15, *p* ≤ 0.05), with RHA rats globally showing higher levels compared to RLA rats (Figure 3A,B). Moreover, the analysis revealed significant effects of NH on BDNF levels in both the PFC (F(1,33) = 36.8, *p* < 0.001; Figure 3A,B) and ACg (F(1,34) = 6.5, *p* = 0.016; Figure 4A,B), indicating an overall increase of BDNF levels due to NH. Additionally, there was a “Strain x NH” interaction effect on BDNF levels in the ACg (F(1,34) = 6.4, *p* = 0.017), as the increasing effect of NH was due to a significant effect in RLA rats (see Duncan’s test in Figure 4B).

There were no main effects nor interactions on trkB levels in PFC or ACg (all F < 2.6, *p* > 0.12, df 1, 34–36; see Figure 3C,D, and Figure 4C,D, respectively).

As for PSA-NCAM levels, ANOVA yielded a nearly significant NH effect (F(1,36) = 4.1; *p* = 0.051; Figure 3E,F) in the PFC, and a significant NH effect on PSA-NCAM in ACg (F(1,36) = 6.0, *p* < 0.02; Figure 4E,F), indicating that NH treatment generally increased the PSA-NCAM levels in both cortical areas (see Duncan’s tests in Figure 3F and Figure 4F).

### 3.6. BDNF, trkB, and PSA-NCAM Protein Levels in vHPC and dHPC of RHA and RLA Rats: Effects of Neonatal Handling

**Figure 5:**
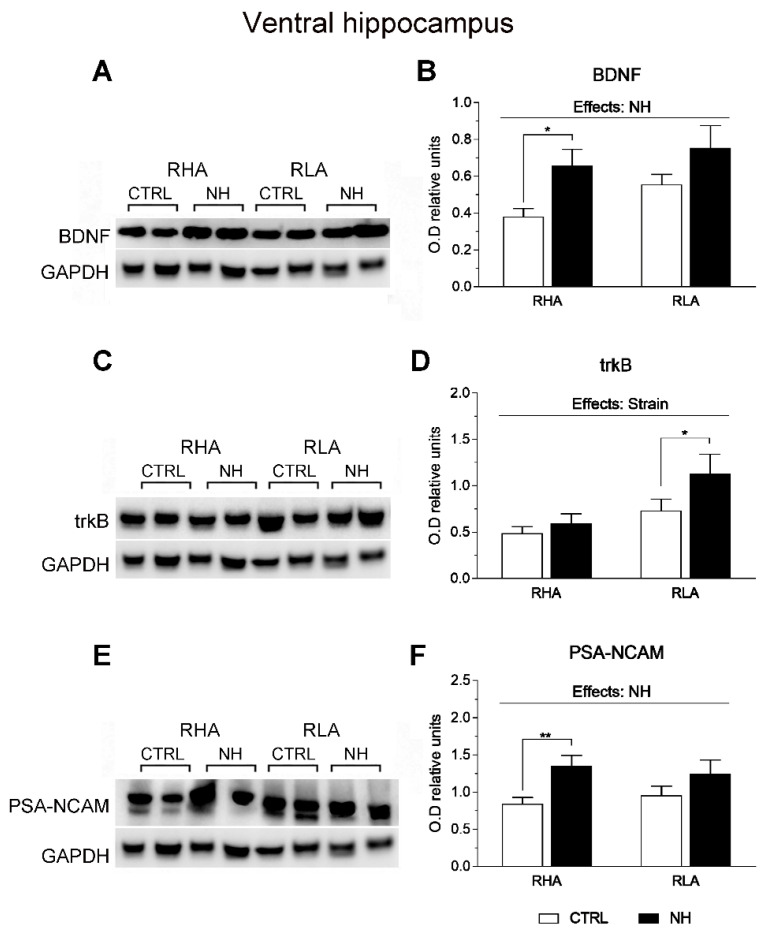
Western blot analysis of the BDNF (**A**,**B**), trkB (**C**,**D**), and the PSA-NCAM (**E**,**F**), in the ventral hippocampus of the RHA and the RLA rats, either untreated controls (CTRL) or treated with neonatal handling (NH). The values represent the densitometric analysis of the BDNF/GAPDH (**B**), trkB/GAPDH (**D**), and the PSA-NCAM/GAPDH (**F**) band grey optical density (O.D.) ratios. The bars denote the mean ± S.E.M. of 9–10 rats, in each experimental group. *: *p* < 0.05; **: *p* < 0.02 (post hoc Duncan’s multiple range test).

The factorial ANOVA revealed significant NH effects on BDNF levels in the vHPC (F(1,35) = 7.8, *p* = 0.008; Figure 5A,B), as well as on PSA-NCAM in this region (F(1,36) = 8.6, *p* = 0.006; Figure 5E,F). The NH effect was more pronounced in RHA than RLA rats, as shown by post hoc Duncan’s comparisons (see Figure 5B,F).

There was also an effect of Strain on trkB levels in the vHPC (F(1,32)= 8.1, *p* = 0.008; Figure 5C,D), indicating the overall higher levels of trkB in RLA rats (Figure 5D).

**Figure 6:**
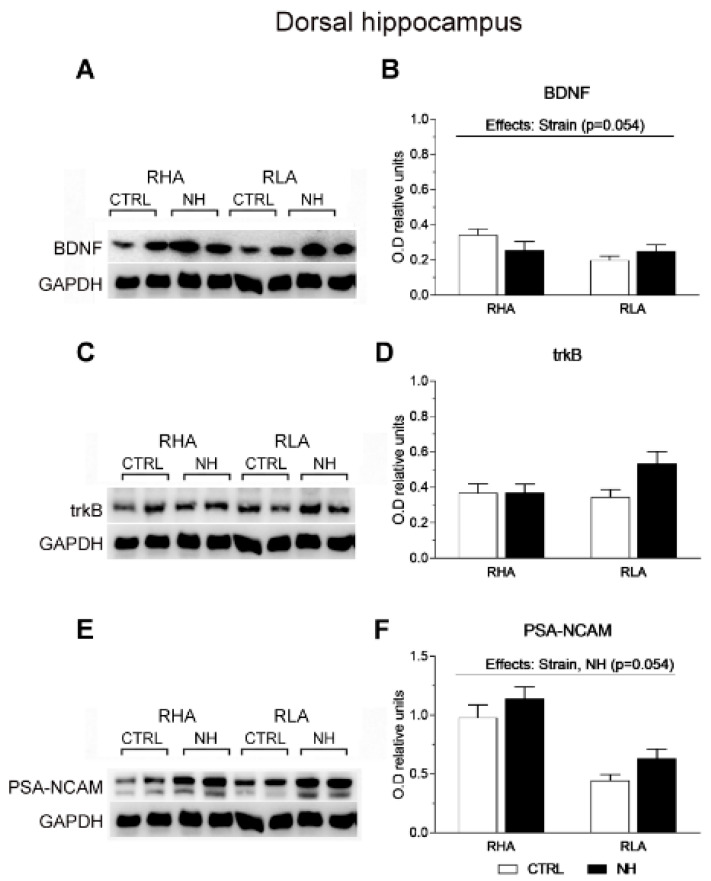
Western blot analysis of the BDNF (**A**,**B**), trkB (**C**,**D**), and the PSA-NCAM (**E**,**F**), in the dorsal hippocampus of the RHA and the RLA rats, either untreated controls (CTRL) or treated with neonatal handling (NH). The values represent the densitometric analysis of the BDNF/GAPDH (**B**), trkB/GAPDH (**D**), and the PSA-NCAM/GAPDH (**F**) band grey optical density (O.D.) ratios. The bars denote the mean ± S.E.M. of 9–10 rats, in each experimental group.

Regarding the dHPC, a nearly significant effect of Strain was found on BDNF levels (F(1,35) = 3.9, *p* = 0.054; Figure 6A,B), due to a trend for higher BDNF levels in RHA rats. There were no significant main factor nor interaction effects on trkB (all F < 3.5, *p* > 0.07; Figure 6C,D). Moreover, ANOVA revealed a significant effect of Strain on PSA-NCAM levels (F(1,36) = 35.6, *p* < 0.001), indicating that RHA rats show markedly higher PSA-NCAM levels than RLAs (Figure 6E,F), and a nearly significant effect of NH (F(1,36) = 3.96, *p* = 0.054), suggesting a trend for increased PSA-NCAM levels in NH-treated rats (Figure 6F).

### 3.7. Correlational Analyses: Relations Among BDNF, trkB, and PSA-NCAM Protein Levels in the Different Areas, and Between Specific Protein Levels and Behaviour, Hormonal, and Cognitive Measures

Concerning correlations among the different protein levels (n = 36–40), the following were observed (see Supplementary Table S1): (1) BDNF in the PFC correlated with PSA-NCAM in the vHPC (r = 0.33, *p* = 0.046) and the ACg (r = 0.45, *p* = 0.005). (2) BDNF levels in the vHPC correlated with BDNF (r = 0.49, *p* = 0.001) and trkB (r = 0.58, *p* < 0.001) in the dHPC, and with trkB (r = 0.58, *p* < 0.001) and PSA-NCAM levels (r = 0.70, *p* < 0.001) in the ACg. (3) BDNF levels in dHPC correlated with trkB in the dHPC (r = 0.37, *p* = 0.02) and PSA-NCAM levels in the ACg (r = 0.38, *p* = 0.017). (4) PSA-NCAM levels in the PFC correlated with trkB levels in the PFC (r = 0.56, *p* < 0.001) and in the vHPC (r = 0.37, *p* = 0.021), as well as with PSA-NCAM levels in the vHPC (r = 0.48, *p* = 0.002). (5) PSA-NCAM levels in the ACg correlated with trkB levels (r = 0.62, *p* < 0.001) in the ACg (Supplementary Table S1).

To highlight the most relevant correlations between protein levels and behavioural variables, Pearsons’ coefficients (n = 35–40) revealed significant results in the NOE test, specifically between BDNF in PFC and “Latency to explore the novel object” (r = −0.41, *p* = 0.01) and “Time spent exploring the novel object” (r = 0.45, *p* = 0.002) (Supplementary Table S2). Additionally, BDNF in the PFC negatively correlated with post-stress corticosterone (r = −0.36, *p* = 0.03) and prolactin (r = −0.37, *p* = 0.03) levels (Supplementary Table S2). Similarly, the “Time spent exploring the novel object” positively correlated with levels of BDNF in the ACg (r = 0.47, *p* = 0.002; Supplementary Table S2) and PSA-NCAM in the vHPC (r = 0.44, *p* = 0.005; Supplementary Table S3).

Remarkably, PSA-NCAM levels in the vHPC were also correlated with the spatial working memory index “T1-T2” (r = 0.42, *p* = 0.008) (Supplementary Table S3).

### 3.8. Exploratory Factor Analysis of Protein Levels, Behaviour, and Stress Hormone Values

A varimax-rotated factor analysis (Varimax orthogonal rotation), including all proteins and brain areas, yielded three independent factors (Table 2A). From this three-factor solution, the two protein measures with the highest loadings on each of the three factors were selected for further analysis. Thus, the selected proteins were trkB in the ACg and BDNF in the vHPC (first factor), PSA-NCAM in the PFC and PSA-NCAM in the vHPC (second factor), BDNF in the PFC and PSA-NCAM in the dHPC (third factor) (see Table 2A). These six protein measures were used for further factor analysis including also the selected behavioural–hormonal variables, i.e., NOE-T, “Total %PPI” (averaged for the four prepulse intensities), the “T1–T2” spatial working memory index (DMTP task), and post-stress corticosterone levels.

We then carried out an exploratory obliquely rotated (oblimin direct rotation) principal components analysis to study associations among protein levels, behavioural measures, and post-stress hormone values. This analysis led to three independent (uncorrelated) components (Table 2B). “Component 1”, tentatively named “Attention-Stress”, is formed by high loadings of PSA-NCAM-dHPC (0.84), “Total % PPI” (−0.74) and post-stress corticosterone (−0.79), as well as low-moderate loading (−0.51) of the DMTP-“T1–T2” working memory index (Table 2B). Moreover, the associations observed among the variables included in Component 1 are generally in agreement with the correlational patterns among these measures (as shown in Supplementary Tables S2 and S3). “Component 2”, tentatively named “Emotion-Memory”, constitutes a summary of the observed effects of NH on BDNF-PFC (loading 0.52), PSA-NCAM- PFC (loading 0.74), and PSA-NCAM-vHPC (loading 0.78) levels, which are all increased by NH in both rat strains, and NOE-T (loading 0.73) and DMTP-“T1–T2” (loading 0.56 ), which are also increased by NH in both rat strains. Moreover, the associations among the variables included in Component 2 generally agree with the correlational patterns observed among these measures (as shown in Supplementary Tables S2 and S3). “Component 3” only grouped trkB-ACg and BDNF- vHPC levels (Table 2B).

## 4. Discussion

In the present study, we report for the first time the long-lasting effects of NH on BDNF, trkB, and PSA-NCAM levels in the PFC, ACg, vHPC, and dHPC of adult RHA and RLA rats. The main results show (1) anxiety-related behaviours and stress-hormone responses were generally reduced by NH in both rat strains; (2) sensorimotor gating, measured through PPI, was improved by NH in RHAs, particularly at prepulse intensities of 65–70 dB, while showing a slight (non-significant) impairment in RLA rats; (3) spatial working memory, assessed through a DMTP task, was significantly improved in RHA rats; (4) BDNF and PSA-NCAM levels increased after NH in the PFC, ACg, and vHPC of both rat strains; (5) strain-dependent effects were observed only on BDNF levels in the PFC (RHA > RLA), PSA-NCAM levels in the dHPC (RHA > RLA), and trkB levels in the vHPC (RHA < RLA).

The enhanced novelty-induced behavioural inhibition (NOE), anxiety (EZM), baseline startle, and stress-induced increments on plasmatic levels of corticosterone and prolactin in control RLA rats, along with the poorer PPI and spatial working memory observed in untreated RHAs, corroborate previous reports (e.g., [5,6,11,12,63]; see reviews by [2,3,9,18,21], and references therein). It must be said that no “cued” navigation (control) task was carried out here, given that we have consistently found no differences between RHA and RLA rats in such a task in previous studies (e.g., reviewed by [11]). Regarding the NH intervention, the current findings related to behavioural/cognitive (anxiety-related, PPI, and working memory) and stress hormone responses are also consistent with prior findings on NH effects, in both RLA and RHA rats (e.g., reviewed by [12,44]), as well as in non-selected laboratory rat strains (e.g., [44,45,46,47,48,49,50,51,52,53,54,55,59,60,61,64]).

Previous reports have shown increased BDNF levels, along with reduced anxiety and/or attenuated stress hormone responses, and/or improved memory, following NH. However, these studies mainly focused on the HPC and used laboratory rat strains that were not selected for any specific behavioural trait [55,59,60]. Furthermore, one study by [61] reported that NH had a stimulatory effect on hippocampal PSA-NCAM expression in aged Wistar rats. Notably, the main novelty of the present study is that NH induced very long-lasting enhancements of BDNF and PSA-NCAM levels in the PFC, ACg, and vHPC in two genetically selected strains, the RHA and RLA rats, which differ in their anxiety-related behaviour, coping style, stress hormone responses, attention and sensorimotor gating, cognition, and other neurobehavioural traits associated with vulnerability to stress/depression and schizophrenia-linked features [2,3,4,5,6,9,10,11,12,13,14,17,18,19,20,21]. These neuroplastic/synaptic effects of NH were paralleled by important reductions of anxiety-related behaviour, especially in RLAs, as well as with improvements in PPI and spatial working memory in RHA rats. Additionally, both rat strains showed overall reductions in stress-hormone responses.

Compelling morphological, functional, neurochemical, and gene-expression evidence indicates that the frontal cortex (FC; including the ACg), the PFC, and the HPC show different profiles in RHA vs. RLA rats. In fact, most findings are consistent with the notion that RHA rats exhibit impaired maturation and decreased function of the FC, PFC, and the HPC (e.g., [2,12,16,18,65]). Of note in this context, the medial PFC and the ACg are linked—among other functions—to cognitive control and emotional impulse control, planning and executive function, top-down inhibitory control, working/active memory, memory retrieval and interference, as well as managing negative emotions related to pain and frustration (e.g., [7,66,67,68,69,70]). Moreover, the HPC plays a crucial role in detecting goal-conflicts, such as approach-avoidance conflict, and is the core component of the “goal inhibition system”, which is anatomically based in the septo-hippocampal system (SHS; [69]). The SHS operates as a “comparator” for goal conflict detection resolution, interconnecting with the above regions and the amygdala (AMY), and controlling conflict-induced anxiety and outputs linked to the AMY, such as freezing behaviour or stress-induced hormone responses, while also managing “conflicting” memory interference (e.g., see reviews by [68,69]). In this context, alterations in the PFC and the HPC have been implicated in various psychopathological conditions, including anxiety, depression, schizophrenia, and disorders related to impulse control, such as addiction.

It is noteworthy that the Roman strains exhibit two markedly divergent phenotypes. RHA rats are characterized by their resistance to stress and depression, while exhibiting impulsivity, schizophrenia-relevant sensorimotor gating and attentional/cognitive deficits, and a vulnerability to drug addiction, placing them within the “externalizing” symptom spectrum (reviewed by [2,3,4]). Conversely, RLA rats are behaviourally inhibited, especially under situations involving goal conflict or approach-avoidance conflict; moreover, they tend to be anxious, frustration-prone, stress- and depression-prone, and are considered a valid model of the “internalizing” psychopathological spectrum (e.g., [2,3,4]). Substantial neurobiological, pharmacological, molecular, and behavioural evidence compels the distinction between the two strains (e.g., [2,3,4,8,9,10,11,12,13,14,15,16,17,18,19,20,21,44,63,65]), and supports their validity as models for externalizing (RHA) or internalizing (RLA) psychopathology-related symptoms [3].

Importantly, NH enduringly and positively influenced the genetically based behavioural/attentional/cognitive profiles of RHA and RLA rats, and also enhanced baseline neuroplasticity markers such as BDNF and PSA-NCAM in the PFC, ACg, and HPC. Of note in this context, the BDNF/trkB system plays a crucial neurotrophic role by supporting neuronal viability, regulating dendritic and axonal morphology, and affecting synaptogenesis and synaptic transmission [71]. PSA-NCAM is involved in neuroplastic events, including the regulation of dendrite and spine/synapse numbers (synaptogenesis and synapse survival), and it facilitates the BDNF/trkB signalling (reviewed by [14,15]). Moreover, decreases in BDNF and PSA-NCAM, at least in the HPC, appear to be linked to stress conditions and depression, whereas chronic antidepressant treatments enhance the levels of both molecules (reviewed by [14,15]). In this context, the most consistent and clear effects of the NH intervention in rats include an enduring reduction in anxiety and behavioural/hormonal reactivity to stressors, increased resistance to stress-induced depression-like behaviour (e.g., learned helplessness, forced swimming test), improved coping abilities in aversive and approach-avoidance conflict situations, increased playfulness, and improved functions of the HPC (e.g., [12,44,45,46,49,50,51,52,55,64,72,73,74,75] and present results). Therefore, it is reasonable to assume that the NH effects reported herein on BDNF and/or PSA-NCAM in the PFC, ACg, and vHPC reflect “positive” neuroplastic synaptic processes in these regions. This, in turn, could explain the observed decreases in anxiety measures and hormonal responses to stress, and the improvements in PPI and working memory. Interestingly, significant positive correlations (as shown in Supplementary Table S2) were found between BDNF in PFC and behaviour in the NOE test, along with negative correlations between BDNF in the PFC and post-stress corticosterone and prolactin levels. Similarly, the “Time spent exploring the novel object” in the NOE was positively correlated with BDNF levels in the ACg (Supplementary Table S2) and with PSA-NCAM levels in the vHPC (Supplementary Table S3). Since there is a consistent positive correlation (r = 0.56, *p* < 0.001, n = 84) between object exploration in the NOE test and the “Number of head dips” (anxiety index) in the EZM test (see also [12]), these correlations indicate a negative relationship between anxiety and BDNF levels in the PFC and ACg, as well as PSA-NCAM levels in the vHPC. Importantly, PSA-NCAM in the vHPC had a positive correlation with the spatial working memory index “T1-T2” (see Supplementary Table S3). Moreover, these correlations are generally consistent with the results of the principal components factor analysis (PCA) including the main protein values (selected through a previous orthogonally rotated factor analysis; see Table 2A) and behavioural variables. In fact, in line with the observed correlations, the first component of the PCA analysis (explaining 25.4% variance) shows associations of PSA-NCAM in the dHPC (loading 0.84) with “Total %PPI” (loading −0.74) and post-stress corticosterone (loading −0.79) (See Table 2B). Also in line with the correlations, the second component of the PCA analysis (explaining 22.5% variance) shows positive associations among BDNF in the PFC (loading 0.52), PSA-NCAM in the PFC (loading 0.74), PSA-NCAM in the vHPC (loading 0.78), “Time spent exploring the novel object” in the NOE test (loading 0.73), and the spatial working memory index “T1–T2” (loading 0.56) (see Table 2B). Thus, both the correlational and principal components analyses suggest that at least some of the neuroplastic/synaptic changes elicited by NH in the PFC and/or HPC may be associated to the enduring emotional and cognitive effects of the neonatal treatment.

In addition, it is noteworthy that the brain regions studied appear to exhibit a sort of “concerted” global and persistent neuroplastic adaptation to NH, as indicated by the increased levels of BDNF (in PFC, ACg and vHPC) and PSA-NCAM (in PFC, ACg, vHPC and dHPC). The notion of a “concerted” neuroplastic effect of NH, is supported by the pattern of significant positive correlation coefficients among the content of different proteins and different brain regions (see Supplementary Table S1). Specifically, the following positive correlations were observed: 1) BDNF in PFC with PSA-NCAM in vHPC and ACg. 2) BDNF in vHPC with BDNF and trkB in dHPC, and with trkB and PSA-NCAM in ACg. 3) BDNF in dHPC with BDNF in vHPC, trkB in dHPC and PSA-NCAM in ACg. 4) PSA-NCAM in PFC with trkB in PFC and vHPC, and with PSA-NCAM in vHPC. 5) PSA-NCAM in ACg with trkB in ACg, and with BDNF in PFC, vHPC and dHPC. 6) PSA-NCAM in the vHPC with the same protein in PFC and BDNF in the PFC (see Supplementary Table S1, and “Results”). These global effects of NH (except the lack of effects on trkB; see below) are not surprising, given the functional interconnectivity among the PFC, ACg, and the HPC (e.g., [66,68], reviewed by [69]). On the other hand, the trkB protein has been shown to respond differently to an acute stressor challenge, depending on both the brain region and the rat strain (e.g., [14,15]). Thus, in the current study, since no acute stress challenge was present (i.e., the effects of NH were evaluated under baseline conditions), it is plausible that the lack of long-term treatment effects on trkB in any of the studied brain regions may be due to compensatory/stabilizing mechanisms within the BDNF/trkB system. Such mechanisms may be triggered by the neurodevelopmental effects of NH on BDNF content and/or function. Further studies designed similarly to the present one but also including a final acute stress challenge of the subjects are warranted to test this hypothesis.

It is noteworthy that despite NH elicited neuroplastic changes on both rat strains, the interaction “strain x NH” (along with post hoc comparisons) revealed that NH significantly enhanced the BDNF levels in the ACg mainly in RLA rats. Furthermore, the post hoc comparisons revealed that NH’s effects on BDNF and PSA-NCAM levels in the vHPC were more pronounced in RHA than RLA rats. These findings in the vHPC might be important given the molecular and morphological data indicating that the HPC is less functional in RHA vs. RLA rats (e.g., [17,18]; reviewed by [2,3,4]). This is further supported by the poorer performance of RHA vs. RLA rats in hippocampus-dependent spatial learning or approach-avoidance conflict tasks (e.g., [2,3,4,11,65]). These specific effects on BDNF and PSA-NCAM in the vHPC of RHA rats would also be compatible with the observation that NH improves PPI and spatial working memory only in this rat strain (see also [12]).

Since the NH procedure involves brief periods of maternal separation (MS), this has led to study whether NH produces stress in the pups. In fact, it is known, since the early NH studies in the 1960s–1970s, that such a brief maternal separation produces mild stress-hormone responses in the pups, particularly after the second week of life, i.e., after postnatal day 14 (see reviews by, e.g., [44,46] and references therein). In addition, maternal separation (MS) leads to changes in rodents’ maternal behaviour, and hence the question of whether these MS-induced variations in maternal behaviour might underlie the effects of NH. In this context, both NH and brief MS (of equal duration as the maternal separation involved in the “neonatal handling” procedure), have been reported to enhance maternal care in rats to a similar extent (reviewed and discussed by [12]). Importantly, however, only NH (but not MS) produced enduring reductions of both hormonal responses to stress and anxiety behaviour in adult rats. This finding means that the enhanced maternal care, due to MS involved in the “neonatal handling”, would not be the principal factor explaining the long-lasting effects of NH intervention. Thus, other aspects of the NH process (such as gently stroking the pups, and/or pup isolation for a few minutes every day) may likely underlie its enduring behavioural and neural consequences (reviewed and discussed by [12]; see also [44,46] and references therein).

## 5. Conclusions

To the best of our knowledge, this is the first report on the permanent neuroplastic changes induced by NH on the content of BDNF and/or PSA-NCAM proteins in PFC, ACg, and HPC in both RHA and RLA rats. In parallel, NH led to reductions in anxiety-related behaviour and stress hormone responses in both rat strains along with improvements in PPI and spatial working memory in RHA rats. It is reasonable to hypothesize that the neuroplastic changes observed in these three brain regions may contribute, at least in part, to the behavioural/cognitive and/or stress hormone responses reported herein. In particular, the more significant NH effects on BDNF and PSA-NCAM in the vHPC of RHA rats might contribute to their improved performance in PPI and spatial working memory tasks.

Due to space and breeding restrictions (since we had to keep the females for breeding purposes in order to maintain our colony of Roman rats), it was not possible to include females in the present studies. In this regard, while NH treatment effects have primarily been validated using male rats (as can be seen in the above referenced NH literature), when females have been used or compared with males, gender effects (and/or “sex x NH” interactions) have often been observed, including the Roman rats (e.g., [18,21,48,53,63]). Therefore, it would be relevant to include females of both rat strains in future studies, to compare the effects of NH between both sexes and to explore the generalizability of the present findings.

In conclusion, further investigation is warranted to shed light on the mechanisms involved in the relationship between the molecular neuroplastic effects and behavioural changes elicited by NH; nevertheless, the present findings add experimental support to the view that the synaptic structural and/or functional plastic changes induced by BDNF/trkB and PSA-NCAM during both early life and adulthood may play a key role in the experience-dependent behavioural and cognitive effects of NH (e.g., [12,55,59,60,61,76]).

## Figures and Tables

**Table 1:** Means (± SEM) of: **NOE-L**; latency (s) to the first exploration of the novel object in the NOE test. **NOE-T**; total time (s) spent exploring the novel object in the NOE test. **EZM-T**; time spent in the open sections of the EZM test. **EZM-HD**; number of head-dips through the border of the open section of the EZM test. **Baseline startle**; mean startle response for the first 10 pulse-alone trials of the session. **%PPI**; percentage prepulse inhibition at the different prepulse intensities (65 dB–80 dB). **Total %PPI**; percentage prepulse inhibition averaged for the four prepulse intensities. **DMTP task [mean (T1–T2)]**; subtraction of the distance travelled (cm) to reach the hidden platform in the “odd (T1) minus even (T2) trials”, averaged for the three training days, of the delayed-matching-to-place (spatial working memory) task in the Morris water maze. **“CORT”, “PRL”;** corticosterone and prolactin plasmatic levels, either at “Baseline” or after stress (post-stress) administration. The “n” for each experimental group and for each test is given in parenthesis under the mean (SEM) values. PND; means postnatal day. S, NH, S*NH; Strain, NH or "Strain * NH" significant effects from ANOVA. **a**, *p* < 0.05 vs. the respective control (CTRL) group of the same rat strain. **b**, *p* < 0.05 vs. RLA-CTRL group. All comparisons with Duncan’s multiple range test following significant ANOVA effects. **c**, *p* < 0.05 vs. RLA-CTRL group (Student’s *t*-test). **(#);** the EZM test is from a separate study (2nd BATCH) with male RHA and RLA rats, that were previously tested for NOE at PND60, and was carried out during the same period as the “MAIN BATCH” study. **(##);** these values correspond to a separate DMTP study that was carried out during the same period, using only control (untreated; CTRL) RHA and RLA males, which reflects the typical between-strain difference (RHA < RLA; Student’s *t*-test) in the “spatial working memory” index, as has been observed in several independent studies (see, e.g., [2,11]). **(&)**; a pairwise comparison (Student’s *t*-test) was performed between the RLA-CTRL and RHA-CTRL groups (which, as indicated, is significant) because according to results from previous studies, it was expected that the mean “Baseline startle” response would be lower in RHA-CTRL than in RLA-CTRL rats (e.g., see [11]).

**Anxiety-related and stress hormone responses, PPI and spatial working memory (DMTP) in control (untreated; CTRL) and neonatally-handled (NH) Roman high- (RHA) and low-avoidance (RLA) rats.**					
	**Means (±SEM)**				
**“MAIN BATCH”**	RLA-CTRL	RLA-NH	RHA-CTRL	RHA-NH	EFFECTS
**NOE-L** (PND60)	61.8 (12.5) (n = 20)	8.5 (1.3) **a** (n = 20)	10.9 (3.8) **b** (n = 20)	4.4 (1.3) **a** (n = 22)	S, NH, S*NH
**NOE-T**	19.7 (6.6)	112.6 (5.9) **a**	93.0 (5.8) **b**	125.2 (9.5) **a**	S, NH, S*NH
**(#) “2nd BATCH”**					
**(#) NOE-L**	49.1 (8.9)	10.2 (1.7) **a**	4.1 (0.7) **b**	3.6 (0.5)	S, NH, S*NH
**(#) NOE-T** (PND60)	13.4 (2.7) (n = 18)	105.0 (6.4) **a** (n = 22)	121.1 (3.2) **b** (n = 22)	142.9 (3.0) **a** (n = 22)	S, NH, S*NH
					
**(#) EZM-T**	68.0 (9.1)	103.7 (9.6) **a**	83.3 (9.7)	144.2 (5.6) **a**	S, NH
**(#) EZM-HD** (PND90)	6.9 (0.7) (n = 18)	9.8 (0.8) **a** (n = 22)	12.8 (0.8) **b** (n = 22)	13.8 (0.7) (n = 22)	S, NH
**“MAIN BATCH”**					
**Baseline startle (&)** (PND100)	2265.5 (418.8) (n = 16)	1915.6 (295.6) (n = 18)	1301.3 (214.3) **b** (n = 14)	1806.7 (275.5) (n = 16)	
**% PPI**					
65dB	42.3 (7.5)	30.3 (5.3)	12.0 (6.8) **b**	33.4 (8.4) **a**	S
70 dB	53.6 (5.1)	50.1 (5.2)	8.2 (8.3) **b**	38.9 (10.2) **a**	NH*Intensity
75 dB	67.5 (4.7)	56.2 (5.0)	41.2 (5.3) **b**	52.1 (7.8)	
80 dB	72.2 (3.7)	65.0 (3.5)	52.4 (6.1) **b**	66.1 (4.2)	
					
**Total %PPI**	58.9 (4.4)	50.6 (4.2)	28.5 (5.2) **b**	47.8 (7.2) **a**	S, S*NH
**DMTP task [mean (T1-T2)]** (PND140)	1229.0 (232.8) (n = 16)	1542.1 (211.6) (n = 18)	839.4 (404.8) (n = 14)	1877.3 (422.9)**a** (n = 16)	NH
**(##)**	1625.4 (308.7) (n = 10)		252.4 (674.6) **c** (n = 12)		
**BASELINE** **CORT** (ng/mL) (PND210)	66.4 (11.3)	46.1 (5.8)	73.9 (9.1)	65.6 (8.6)	
**POST-STRESS** **CORT** (ng/mL)	329.2 (16.5) (n = 8)	311.9 (21.4) (n = 8)	265.1 (10.9) **b** (n = 10)	210.9 (14.5) **a** (n = 9)	S, NH
**BASELINE PRL** (ng/mL) (PND210)	14.4 (3.0)	16.1 (3.6)	6.0 (0.9)	9.8 (1.1)	S
**POST- STRESS PRL** (ng/mL)	33.1 (5.0) (n = 6)	22.6 (1.8) **a** (n = 8)	13.6 (0.9) **b** (n = 7)	9.5 (1.5) (n = 9)	S, NH

**Table 2:** (**A**) **Varimax-rotated (orthogonal) factor analysis including the 12 protein measures.** From this varimax-rotated factor analysis, the two molecular parameters (in bold) with the highest loadings in each factor were selected for further principal components analysis. (**B**) **Obliquely rotated (oblimin direct) principal components factor analysis.** The “oblimin direct” rotation shows three components (or factors) that are independent, as correlations between factors are 0.062 (1 with 2), 0.004 (1 with 3), 0.029 (2 with 3). Included are loadings **> 0.50** (absolute value). Total N= 36 in both analyses (n = 8–10 per experimental group; because only rats having values for all measures are included in both analyses).

**(A) Varimax-rotated (orthogonal) factor analysis with the twelve protein parameters**			
	**COMPONENTS**		
	**1**	**2**	**3**
BDNF-PFC	0.18	0.40	**0.64**
trkB-PFC	−0.23	0.49	0.49
PSA-NCAM-PFC	−0.06	**0.82**	−0.08
BDNF-ACg	0.50	−0.17	0.50
trkB-ACg	**0.85**	−0.16	0.19
PSA-NCAM-ACg	0.67	0.09	0.44
BDNF-vHPC	**0.83**	−0.08	−0.05
trkB-vHPC	0.02	0.47	−0.63
PSA-NCAM-vHPC	0.30	**0.76**	0.09
BDNF-dHPC	0.30	−0.68	−0.03
trkB-dHPC	0.80	0.11	−0.19
PSA-NCAM-dHPC	−0.12	0.04	**0.72**
% variance explained	**29.03**	**19.65**	**14.20**
Total variance explained	**62.9%**		
**(B) Obliquely rotated (oblimin direct rotation) principal components factor analysis with the selected molecular, behavioural, and hormonal measures**			
	**COMPONENTS** **(“Tentative names”)**		
	**1**	**2**	**3**
	**“Attention-stress”**	**“Emotion-memory”**	
BDNF-PFC	---	**0.52**	---
PSA-NCAM-PFC	---	**0.74**	---
trkB-ACg	---	---	**0.86**
BDNF-vHPC	---	---	**0.83**
PSA-NCAM-vHPC	---	**0.78**	---
PSA-NCAM-dHPC	**0.84**	---	---
NOE-T	---	**0.73**	---
% PPI	**−0.74**	---	---
DMTP-WM	**−0.51**	**0.56**	---
Post-Stress-Cort	**−0.79**	---	---
% explained variance	25.4	22.5	17.6
Total variance (%)	65.5%	---	---

## Data Availability

The data presented in the current study are available from the corresponding authors on reasonable request. The data are not publicly available due to privacy restrictions.

## Supplementary Materials

The following supporting information can be downloaded at: https://www.mdpi.com/article/10.3390/brainsci15080776/s1, Table S1: Pearsons’ correlation coefficients among the different protein values in different brain areas; Table S2: Pearsons’ correlation coefficients among PFC and ACg protein levels and behavioural-hormonal variables; Table S3: Pearsons’ correlation coefficients among vHPC and dHPC protein levels and behavioural-hormonal variables.
