# Supplementary material for: Neonatal Handling Positively Modulates Anxiety, Sensorimotor Gating, Working Memory, and Cortico-Hippocampal Neuroplastic Adaptations in Two Genetically Selected Rat Strains Differing in Emotional and Cognitive Traits
Source: Brain Sci. 2025 Jul 22;15(8):776. doi: 10.3390/brainsci15080776 (PMC12384143; doi:10.3390/brainsci15080776)
Supplement: Supplementary file 1 [file brainsci-15-00776-s001.zip › brainsci-3715669-supplementary.pdf]

Supplementary Table 1.- Pearsons' correlation coefficients among the different protein values in different brain areas

	(1)	(2)	(3)	(4)	(5)	(6)	(7)	(8)	(9)	(10)	(11)	(12)
(1) BDNF-PFC	**1**											
(2) trkB-PFC	-.22	**1**										
(3) PSA-NCAM-PFC	-.01	**.56*** **	**1**									
(4) BDNF-ACg	.16	**-.62*** **	-.15	**1**								
(5) trkB-ACg	.31	**-.36***	-.31	.26	**1**							
(6) PSA-NCAM- ACg	**.45** **	-.06	-.12	.02	**.62*** **	**1**						
(7) BDNF-vHPC	.25	-.11	-.21	.03	**.58*** **	**.70*** **	**1**					
(8) trkB-vHPC	-.18	**.39***	**.37***	-.14	-.28	**.48** **	-.13	**1**				
(9) PSA-NCAM-vHPC	**.33***	.26	**.48** **	.09	.16	.30	.18	.14	**1**			
(10) BDNF-dHPC	-.07	-.01	**-.34***	-.07	.29	**.38***	**.49*** **	-.30	-.28	**1**		
(11) trkB-dHPC	.24	.01	-.06	.04	**.56*** **	**.54*** **	**.58*** **	.34	.16	**.37***	**1**	
(12) PSA-NCAM-dHPC	.27	.18	.23	.03	-.04	.11	-.19	-.20	.11	.16	-.18	**1**

N=36-40 (including the rats from the 4 experimental groups; $n \geq 9$ per group). Abbreviations: BDNF, trkB and PSA-NCAM in the prefrontal cortex (PFC), anterior cingulate cortex (ACg), ventral hippocampus (vHPC) and dorsal hippocampus (dHPC). * $p<0.05$; ** $p<0.01$; *** $p<0.001$ (two tailed).

Supplementary Table 2.- Pearsons' correlation coefficients among PFC and ACg protein levels and behavioural-hormonal variables

	(1)	(2)	(3)	(4)	(5)	(6)	(7)	(8)	(9)	(10)	(11)	(12)	(13)
(1) BDNF-PFC	**1**												
(2) trkB-PFC	-.22	**1**											
(3) PSA-NCAM-PFC	-.01	**.56*****	**1**										
(4) BDNF- ACg	.16	**-.62*****	-.15	**1**									
(5) trkB- ACg	.31	**-.36***	-.31	.26	**1**								
(6) PSA-NCAM- ACg	**.45****	-.06	-.12	.02	**.62*****	**1**							
(7) NOE-L	**-.41***	-.20	-.26	.15	.14	-.11	**1**						
(8) NOE-T	**.45****	.17	.31	**.47****	.03	.09	**-.54*****	**1**					
(9) BASELINE STARTLE	**.33***	-.11	-.17	-.09	.25	.11	.67	.08	**1**				
(10) %PPI (averaged for the entire session)	-.08	-.02	-.16	.04	.28	-.07	**.41***	.01	**.44****	**1**			
(11) DMTP-"T1 – T2"	.09	.08	.30	.15	-.06	-.01	.01	**.33***	.03	.23	**1**		
(12) POST STRESS- CORT	**-.36***	.12	-.05	-.16	.06	.05	**.37***	.01	-.03	**.39***	.23	**1**	
(13) POST STRESS- PRL	**-.37***	-.07	-.13	-.12	.25	.00	**.41***	-.12	.22	**.39***	.20	**.56*****	**1**

	(1)	(2)	(3)	(4)	(5)	(6)	(7)	(8)	(9)	(10)	(11)	(12)	(13)
(1) BDNF-vHPC	**1**												
(2) trkB-vHPC	-.13	**1**											
(3) PSA-NCAM-vHPC	.18	.14	**1**										
(4) BDNF-dHPC	**.49*****	-.30	-.28	**1**									

N=35-40, including the 4 experimental groups (n ≥ 8-10 per experimental group), except for correlations with Post-stress prolactin, in which N=30 (n ≥ 6-9 per experimental group). * $p<0.05$; ** $p<0.01$; *** $p<0.001$ (two tailed). Abbreviations.- NOE-L, NOE-T; latency to the first exploration of the novel object, and total time spent exploring the object (respectively) in the "novel object exploration" (NOE) test. BASELINE STARTLE; averaged startle response during the first 10 trials (pulse alone) of the "startle + PPI" test session. %PPI; percentage prepulse inhibition averaged for the four prepulse intensities (entire session). DMTP-"T1 – T2"; spatial working memory index (trial 1 minus trial 2, averaged for the three training days). POST STRESS-CORT; POST STRESS-PRL; post-stress plasma levels of corticosterone and prolactin, respectively. Other abbreviations as in Supplementary Table 1.

(5) trkB-dHPC	**.58*****	.34	.16	**.37***	**1**								
(6) PSA-NCAM-dHPC	-.19	-.20	.11	.16	-.18	**1**							
(7) NOE-L	-.04	-.04	-.14	-.15	-.17	**-.35¨**	**1**						
(8) NOE-T	.18	.22	**.44****	-.03	.26	.05	**-.54*****	**1**					
(9) BASELINE STARTLE	-.02	-.14	.06	-.10	.15	-.15	.07	.08	**1**				
(10) %PPI	.06	-.09	-.07	-.21	.19	**-.54*****	**.41***	.01	**.44****	**1**			
(11) DMTP-"T1 – T2"	.01	.15	**.42****	-.20	.11	-.17	.01	**.33***	.03	.23	**1**		
(12) POST STRESS- CORT	.14	.18	.06	-.18	.20	**-.57*****	**.37***	.01	-.03	**.39***	.23	**1**	
(13) POST STRESS- PRL	.09	.27	-.04	-.26	.29	**-.58*****	**.41***	-.12	.23	**.39***	.20	**.56*****	**1**

Supplementary Table 3.- Pearsons' correlation coefficients among vHPC and dHPC protein levels and behavioural-hormonal variables

N=35-40, including the 4 experimental groups (n ≥ 8-10 per experimental group), except for correlations with Post-stress prolactin, in which N=30 (n ≥ 6-9 per experimental group). * p<0.05; **p<0.01; ***p<0.001 (two tailed). Abbreviations as in Supplementary tables 1-2.
